# Retrieval and Evaluation of Chlorophyll-a Concentration in Reservoirs with Main Water Supply Function in Beijing, China, Based on Landsat Satellite Images

**DOI:** 10.3390/ijerph18094419

**Published:** 2021-04-21

**Authors:** Yuequn Lai, Jing Zhang, Yongyu Song, Zhaoning Gong

**Affiliations:** 1 Beijing Key Laboratory of Resource Environment and Geographic Information System, Capital Normal University, Beijing 100048, China; 2200901009@cnu.edu.cn (Y.L.); 2190901014@cnu.edu.cn (Y.S.); 2 Key Laboratory of 3D Information Acquisition and Application of Ministry of Education, Capital Normal University, Beijing 100048, China; gongzhn@cnu.edu.cn; 3 Beijing Laboratory of Water Resources Security, Beijing 100048, China; 4 Key Laboratory of Mechanism, Prevention and Mitigation of Land Subsidence, MOE, Beijing 100048, China

**Keywords:** chlorophyll-a, stepwise regression model, remote sensing technique, nutritional status evaluation

## Abstract

Remote sensing retrieval is an important technology for studying water eutrophication. In this study, Guanting Reservoir with the main water supply function of Beijing was selected as the research object. Based on the measured data in 2016, 2017, and 2019, and Landsat-8 remote sensing images, the concentration and distribution of chlorophyll-a in the Guanting Reservoir were inversed. We analyzed the changes in chlorophyll-a concentration of the reservoir in Beijing and the reasons and effects. Although the concentration of chlorophyll-a in the Guanting Reservoir decreased gradually, it may still increase. The amount and stability of water storage, chlorophyll-a concentration of the supply water, and nitrogen and phosphorus concentration change are important factors affecting the chlorophyll-a concentration of the reservoir. We also found a strong correlation between the pixel values of adjacent reservoirs in the same image, so the chlorophyll-a estimation model can be applied to each other.

## 1. Introduction

Eutrophication is the result of immoderate growth and reproduction of phytoplankton due to the absorption of excessive nutrients such as nitrogen and phosphorus, which is often the result of human environmental pollution [1]. Eutrophication was not associated with water pollution until the 1930s, but the continuous occurrence of eutrophication worldwide has aroused global attention for its main cause of pollution [2]. Due to the rapid growth in the process of eutrophication and the slow recovery of eutrophic water, the management and treatment of plant growth is difficult and expensive [3,4,5]. Inland lakes are often strongly affected by environmental changes owing to the increasing impact of human activities. It is urgent to monitor the concentration distribution of water quality and the degree of eutrophication, which has great practical value for real-time dynamic monitoring of water quality and emergency treatment of eutrophication and flooding.

Because chlorophyll-a is very significant for photosynthesis, the eutrophication of fresh water and the proliferation of aerobic algae are directly related to the chlorophyll-a (Chl-a) concentration [1]. Many researchers have studied estimation models suitable for different regions [6,7,8,9]. Other scholars have studied the application of different methods to chlorophyll-a remote sensing retrieval. Some researchers have developed or applied algorithms to retrieve water quality parameters in lakes, including chlorophyll-a [10,11]. MODIS data are widely used in the retrieval of chlorophyll-a concentrations in coastal areas because of its wide coverage and high update frequency [12,13,14]. Some scholars have used band ratio and spectral ratio methods to estimate chlorophyll-a concentration [15,16]. Through the sensitivity analysis of AVRIS data and direct analysis of surface-measured hyperspectral data, some scholars determined the best band to estimate chlorophyll-a [15,17]. Xu and Mao, based on GF-1, show that the estimation model based on the logarithmic combination of wavebands can be used to retrieve the chlorophyll-a concentration in the clean water of Qiandao Lake [18]. Landsat-8 and Sentinel-2/3 data have been used for water quality determination owing to their high distribution rate [19,20,21,22,23,24,25]. Feng et al. proposed the use of GF-1 WFV and Landsat-8 OLI to retrieve the chlorophyll-a concentration in Taihu Lake, which can improve the retrieval accuracy [26]. With the development of computer technology, the application of water quality estimation models is increasing, especially in machine learning [27,28,29,30,31,32].

The Guanting Reservoir is an important water source in Beijing, China [33], and is the largest reservoir of the Yongding River. It functions as an important standby water source in Beijing [34], with significant ecological and environmental ties [35,36,37,38]. With the development of the social economy and population growth, the contradiction between the supply and demand of water resources in Beijing is becoming increasingly serious, which has become an obstacle to the sustainable development of the social economy and ecological environment [39,40,41]. The serious water shortage problem in Beijing is mainly related to the actual water storage and net water volume. The annual average outflow of Taihu Lake is approximately 9.468 billion cubic meters [42]. In recent years, the Yongding River has been in a state of no flow, so the water storage is very low. Although the capacities of the reservoir are relatively high, the actual storage capacity is low. Therefore, it is critical to study the distribution, variation, and correlation of chlorophyll-a concentrations in the reservoir for domestic and water use in North China.

The objectives of this study were to (1) monitor the nutritional status of the reservoir in Beijing and ensure the effectiveness and safety of the region’s water resource, (2) discuss the reasons and influences of the change of chlorophyll-a concentration, and (3) analyze the distribution and change of chlorophyll-a concentration in the reservoir to guide water quality supervision and other practices. This study will help relevant departments manage the reservoir, focus on high-risk areas, and control factors that may affect the water quality of the reservoir to help create a good water environment.

## 2. Materials and Methods

### 2.1. Study Area

**Figure 1:**
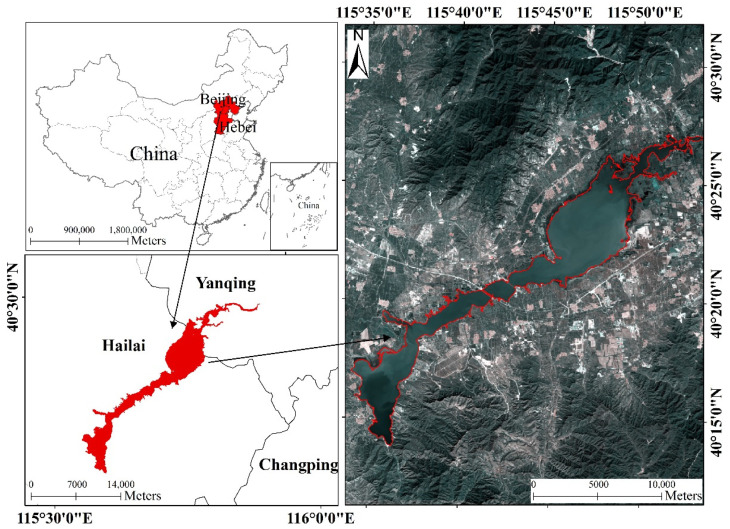
Study area in Guanting Reservoir. The location for Guanting Reservoir is in Huailai County, Zhangjiakou City, Hebei Province, China, and Yanqing County, Beijing City, China. Remote sensing image (30 × 30 m/pixel) is the combination of the band 4, band 3, and band 2 of Landsat-8 in September 2019.

The Guanting Reservoir (40°13′ N–40°25′ N, 115°36′09″ E–115°50′ E) is located in Huailai County, Zhangjiakou City, Hebei Province, China, and Yanqing County, Beijing City, China (Figure 1). This reservoir was the first large reservoir to be built after the founding of new China. The main flow is the Yongding River, Huailai City, Hebei Province, China. The lower reaches of Beijing are often flooded owing to sediment deposition and riverbed elevation. To eradicate the Yongding River, the Guanting Reservoir was built in October 1951 and completed in May 1954. Built at the entrance of the Guanting Gorge, the reservoir has been operating for more than 40 years and plays a key role in flood control, irrigation, and power generation. The Guanting Reservoir is one of the main water supply sources in Beijing. After the 1980s, because of the construction of the Shacheng pesticide plant, the reservoir water has become increasingly polluted by dichlorodiphenyltrichloroethane, known commonly as DDT. During the 1990s, the water quality continued to deteriorate, and in 1997, the reservoir was removed from the urban drinking water system [43,44]. From the beginning of 2005 to the end of 2006, the Guanting Reservoir banned fishing for the first time, allowing the reservoir to provide semi-polluted water. In 2006, the Guanting Reservoir became the fourth standard drinking water source in Beijing after the Miyun, Huairou, and Haizi reservoirs. At present, the water of the Guanting Reservoir is mainly used as an industrial water source in the west of Beijing.

### 2.2. Data Collection

#### 2.2.1. Satellite Data

In this study, five Landsat images (including one Landsat-7 and four Landsat-8 images) were downloaded from the “Geospatial Data Cloud” platform, and Sentinel-2A images (2 July 2016 and 7 July 2017) were downloaded from the European Space Agency to extract the water surface of the Guanting Reservoir and retrieve the chlorophyll-a concentration (Table 1). The selected remote sensing image contains both the Guanting Reservoir and Miyun Reservoir, which is conducive to comprehensive analysis and mutual verification. Owing to the large sampling area, the sampling time is generally 3 days, which is difficult to be consistent with the imaging time. Only remote sensing images with imaging times close to the water quality sampling time were selected. Generally, the sampling time for each year was selected during the summer and the autumn, when the temperature is high and there is no ice in the reservoir. Before or after the rainy season in Beijing, the image quality is clear because of sunny weather and less influence from the cloud layer. Radiation calibration and atmospheric correction were carried out using ENVI software 5.3.

#### 2.2.2. Water Quality Data

**Figure 2:**
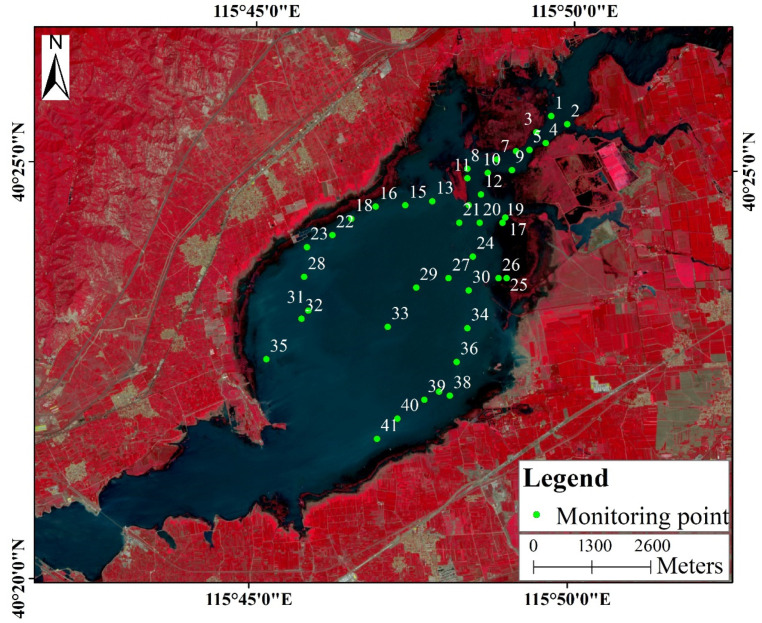
The locations for 41 sampling points are depicted with green circles and numbered 1 to 41 at Guanting Reservoir in 2017. The bottom picture is the standard false color display of the Sentinel-2A image. The sampling points were mainly concentrated in the reservoir area within Beijing, China. The sampling path is along the edge or near the center line of the area.

In this study, water sampling and measurements were conducted in July 2016 and 2017 and September 2019. The geographic location of the sampling vessel was located using a portable global positioning system (GPS). A Hydrolab MS5 (HACH, Loveland, CO, USA) water quality multi-function probe was used. In 2016 and 2017, sampling points were taken along the edge area of the reservoir and across the middle (Figure 2). In 2019, the sampling points were selected in ArcGIS using a fishnet, and the distribution was uniform. The essence of the instrument is to use spectral information and an empirical formula to model and measure. If the probe encounters aquatic plants or other objects underwater, it interferes with the spectrum and produces abnormal values. Abnormal values were eliminated during data processing to obtain effective monitoring points (Table 2). At each sampling point, lake water was collected in clean bottles for further analysis in the laboratory. The parameters selected for measurement included physical parameters (such as turbidity, Sechhi disc transparency, and total suspended solids) and chemical parameters (e.g., Chl-a, hydrogen ion concentration (pH), total nitrogen, total phosphorus, chemical oxygen demand, biochemical oxygen demand, and dissolved oxygen). In this study, we analyzed and evaluated the Chl-a content.

### 2.3. Retrieval Method of Chlorophyll-a Concentration

In this study, the remote sensing images were preprocessed in ENVI software, such as radiometric calibration and atmospheric correction. Then, the water surface boundary was extracted from the remote sensing image by the water body index method, and the reservoir area was cut out in the ENVI software. The values from the image bands of the corresponding location were derived based on the measured data coordinates. In Excel or SPSS software, we performed regression analysis between the bands and its combination values and part of the measured values to obtain the algorithm with the highest correlation as the model for the retrieval of chlorophyll-a concentration. The remaining part of the actual measured value is used for verification. Finally, the verified model was used to estimate the distribution of chlorophyll-a concentration in ENVI software, and the distribution results were displayed by ArcGIS software mapping.

#### 2.3.1. Reservoir Boundary Extraction Method

Based on the remote sensing image, the water areas of the Miyun and Guanting reservoirs were extracted using the index method. In this study, the water area of the Miyun Reservoir was extracted using a decision tree, automatic water extraction index (AWEI) [45], and the improved normalized difference water index (MNDWI) [46,47]. The water area image was cut out from the extracted water surface boundary, and the reflectance value and band calculation value of the corresponding remote sensing image were extracted from the measured point position, correlation analysis was performed to obtain the water quality estimation model, and the chlorophyll-a concentration value of the remaining measured points of the Guanting Reservoir was used for verification. Then, the distribution of chlorophyll-a concentration in the reservoir was obtained by retrieval, and the results were analyzed. The nutritional status of the reservoir was calculated using a modified nutritional status index.

MNDWI = (Green − MIR)/(Green + MIR)(1)

AWEIsh = Blue + 2.5 × Green − 1.5 × (NIR + SWIRI) − 0.25 × SWIR2(2)

where Green is the green band, Red is the red band, Blue is the blue band, NIR is the near-infrared band, MIR is in the mid-infrared band, and SWIR1 and SWIR2 are short-wave infrared bands. The “sh” refers to the formula used when shadow is a major factor.

#### 2.3.2. Chlorophyll-a Concentration Estimation Model

The Landsat-8 OLI has a 30 m resolution and more detailed multi-spectral bands. Sentinel-2A data has four 10 m high-resolution bands. Two types of data were used for retrieval and comparison, and a resolution image suitable for this study was selected.

The empirical model is mainly based on the statistical relationship between chlorophyll-a concentration and remote sensing parameters used to realize the remote sensing retrieval of chlorophyll-a concentration in water. This is a more extensive chlorophyll-a concentration estimation model. It is the most commonly used remote sensing retrieval algorithm for chlorophyll-a concentration to directly calculate the empirical relationship between apparent tourism quantity and water component concentration, based on measured spectrum or water quality parameters and simulated data [48,49]. In this study, the estimation model of the Guanting Reservoir aims to analyze the correlation between the measured parameters and single band, band ratio, normalized difference vegetation index (NDVI) [50], 3BDA-like (Kivu) [51], surface algal bloom index (SABI) [52], and Apple [53] algorithm values of the corresponding Landsat-8 OLI. The best model was obtained to retrieve chlorophyll-a concentration.

SABI, KIVU, NDVI, Apple, and 3-band algorithms are as follows:

Chl_a ∝ SABI = (b5 − b4)/(b2 + b3)(3)

Chl_a ∝ KIVU = (b2 − b4)/b3(4)

Chl_a ∝ NDVI = (b5 − b4)/(b5 + b4)(5)

Chl_a ∝ Apple = b5 − ((b2 − b5) × b5 + (b4 − b5))(6)

Chl_a ∝ 3-band = [1/(b4) − 1/(b5)] × (b6)(7)

where b2, b3, b4, and b5 represent bands 2, 3, 4, and 5 of Landsat-8 OLI, respectively.

#### 2.3.3. Evaluation Indicator

The empirical method is used to construct the model expression between the combination of each band of the Guanting Reservoir remote sensing image and the measured chlorophyll-a concentration. Therefore, based on the predicted and measured values of the model, the linear correlation coefficient (*R*), root mean square error (*RMSE*), and relative root mean square error (*RRMSE*) were used to evaluate the accuracy and stability of the regression model. The calculation methods for *RMSE* and *RRMSE* can be described as follows:

(8) $$R=\frac{\sum \left(x_{i}^{measured}-\bar{x}\right)\left(y_{i}-\bar{y}\right)}{\sqrt{\sum {\left(x_{i}^{measured}-\bar{x}\right)}^{2}\sum {\left(y_{i}-\bar{y}\right)}^{2}}}$$

(9) $$RMSE=\sqrt{\frac{\sum_{i=1}^{N}{\left(x_{i}^{estimated}-x_{i}^{measured}\right)}^{2}}{N}}$$

(10) $$RRMSE=\frac{RMSE}{\sum_{i=1}^{N}x_{i}^{measured}/N}\times 100\%$$

where $\bar{x}$ represents the average value of chlorophyll-a measured, $\bar{y}$ represents the average value of the water surface reflectance on the image, $y_{i}$ is the value of the water surface reflectance on the image, $x_{i}^{estimated}$ represents the simulated value of chlorophyll-a concentration, $x_{i}^{measured}$ represents the measured value of chlorophyll-a concentration, and *N* is the number of test points.

### 2.4. Assessment of Water Nutrition Status

Morihiro Asaki proposed a modified nutritional status index (TSIM) to change the Carlson nutritional status index based on transparency to the chlorophyll-a concentration-based nutritional status index [54,55]. For the Carlson trophic state index or modified trophic state index method, phosphorus is the limiting factor for algal growth. The modified Carlson index uses a continuous value of 0–100 to describe the nutrient status of the lake water. The basic calculation formula is as follows:

TSIm(Chla) = 10 × (2.46 + ln (Chla)/ln (2.5))(11)

where TSIm(Chla) is the modified Carlson index, and Chla is the concentration of chlorophyll-a (μg/L).

## 3. Results

### 3.1. Optimal Estimation Model Selection Results

In this study, 29 chlorophyll-a samples’ data from the Guanting Reservoir for three years (July 2016, July 2017, and September 2019) were selected for correlation analysis with the parameters of single band and multiple band groups of Landsat-8 OLI at corresponding times, as detailed in Table 3. The parameters include the first seven single bands of Landsat-8 OLI, the pairwise ratio of the first five bands, NDVI, KIVU, SABI, Apple, and the widely used 3-band algorithms, with a total of 22 variable values. The correlation of b5 in the single band and b5/b4 in the band ratio is high, and the overall correlation of multiple band combinations is high.

In this study, the measured values of water quality and 22 parameters composed of Landsat-8 OLI bands were analyzed using the stepwise regression method in SPSS 22 software (gradually eliminating the independent variables and improving the correlation coefficient), and a stepwise regression model was obtained. Finally, a group of parameters with high correlation will be selected each year, including a single band, a ratio, and a multi-band parameter value. Then, the linear, polynomial, logarithm, power function, and exponential models were obtained by Excel, and compared with the stepwise regression model by SPSS software (Table 4). This indicates that the stepwise regression model has the best simulation effect, and the correlation can reach more than 89%. These coefficients represent the percentage increase, which varies with the location.

Taking the remaining points of the measured data as verification data, the retrieval accuracy of the stepwise regression model is presented in Table 5. By looking up the critical value table of the linear correlation coefficient, we can see that, when the sample number is 11, the critical value at the significance level of 0.05 is 0.602. As 0.66 is greater than 0.602, the predicted values in 2016 and 2019 are significantly correlated with the measured values at a significance level of 0.05. When the number of samples was nine, the critical value at the level of 0.05 is 0.666, so the predicted value in 2017 was significantly correlated with the measured value at the significance level of 0.05 [56]. Therefore, the three models can be used to retrieve the chlorophyll-a concentration at the corresponding time. The stepwise regression model can retrieve the chlorophyll-a concentration and distribution based on good correlation. Therefore, a stepwise regression model was used to simulate chlorophyll-a concentration in this study.

In the same manner, the measured data, B2, B3, B4, and B5 (distribution represents bands 2, 3, 4, and 8 of Sentinel-2A data, respectively), and their combinations were analyzed by stepwise regression based on Sentinel-2A data, and a stepwise regression model was obtained. Finally, an estimation model of chlorophyll-a concentration in the Guanting Reservoir was developed (Table 6).

### 3.2. Comparison of Retrieval Results Based on Two Image Sources

**Figure 3:**
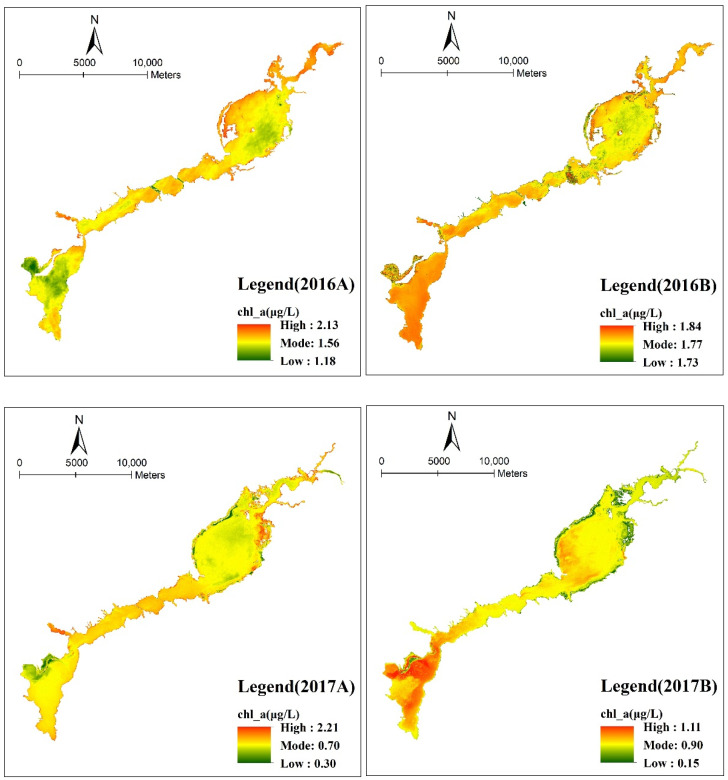
Distribution of chlorophyll-a concentrations of Landsat-8 OLI (2016A, 2017A) and Sentinel-2A (2016B, 2017B) in the Guanting Reservoir in 2016 and 2017. The color bar indicates the closeness of the concentration value to the mode. Red indicates values exceeding the mode, while green indicates values below the mode.

Based on Landsat-8 OLI 30 m resolution multispectral band data and Sentinel-2A 10 m resolution band data, the chlorophyll-a retrieval of the Guanting Reservoir was conducted in the same period (Figure 3). In the case of the 30 m resolution Landsat-8 OLI, the chlorophyll-a concentration was 1.18–2.13 μg/L in 2016 and 0.30–2.21 μg/L in 2017. In the case of the 10 m resolution Sentinel-2A, the chlorophyll-a content was 1.78–1.84 μg/L in 2016 and 0.15–1.11 μg/L in 2017. The value range of the reverse performance decreases. Therefore, with the improvement in resolution, the retrieval is more detailed. Because the sampling points are concentrated in the northern part of the reservoir, the reverse performance of the northern part of the reservoir is more detailed than that of the southern area. From the comparison of the southern part of the reservoir, the higher the resolution, the greater the dependence on the measured data. In the absence of measured data, Sentinel-2A data cannot effectively reflect the change law in the southern part of the reservoir. In general, Landsat-8 OLI data can better retrieve the change in chlorophyll-a in the absence of a large amount of measured data.

### 3.3. Chlorophyll-a Concentration Retrieval Results

**Figure 4:**
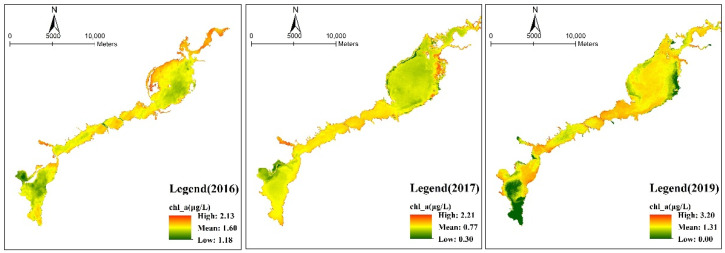
Distribution of chlorophyll-a concentrations in the Guanting Reservoir. The lowest and average values from 2016 to 2017 are declining. From 2017 to 2019, the highest and average values of the Guanting Reservoir are increasing. The color bar indicates the closeness of the concentration value to the mean. Red indicates values exceeding the mean, while green indicates values below the mean.

**Figure 5:**
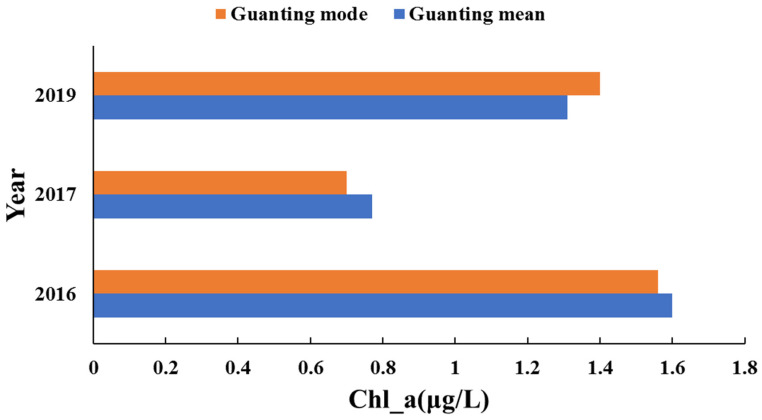
Annual mean and mode changes of chlorophyll-a concentrations in the Guanting Reservoir based on Landsat satellite images. From 2016 to 2017, the concentration of chlorophyll-a in the Guanting Reservoir decreased. In 2019, the concentration slightly increased.

As shown in Figure 4 and Figure 5, the chlorophyll-a content of the Guanting Reservoir shows a downward trend from 2016 to 2017, while the water surface area of the reservoir increased and the water volume was larger in 2019, and the chlorophyll-a content retrieved in some areas was close to 0 μg/L. From the figures of 2016 and 2017, the chlorophyll-a concentration in the narrow and long water areas between the north and south reservoirs of the Guanting Reservoir is higher. Moreover, the chlorophyll-a concentration in the phreatic water area closer to the land was higher. The chlorophyll-a concentration in the northern reservoir area gradually decreased from the central island to the surrounding area. Before 2017, the chlorophyll-a concentration in the southern reservoir gradually increased from the northwest to the dam.

In recent years, the total chlorophyll-a concentration in the Guanting Reservoir was low. After 2016, the overall chlorophyll-a concentration decreased, with an average concentration of 0.97 μg/L. In 2017, the concentration of chlorophyll-a was the lowest, with an average concentration of 0.77 μg/L, which was higher in the southern reservoir than that in the northern area.

### 3.4. Evaluation Results of Water Nutrition Status

**Figure 6:**
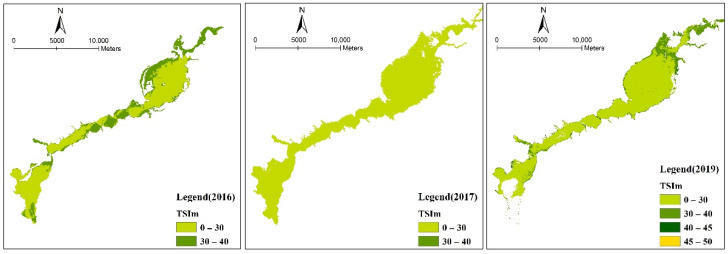
Nutritional status of the Guanting Reservoir. The nutritional index values were mainly concentrated at 0–30, indicating a state of poor nutrition.

According to the nutritional status classification index provided by China’s environmental monitoring, the results show that the nutrient status of the water body in the Guanting Reservoir is gradually improving (Figure 6). The Guanting Reservoir was mesotrophic in 2016, and the reach of the Guishui River into the reservoir and most of the Guishui River reservoir area was mesotrophic. After 2016, the eutrophication status of the reservoir water improved as a whole, and it was in an oligotrophic state. Some areas on the edge of the reservoir were close to the mesotrophic state, resulting in a lack of oxygen in the mean temperature layer. In 2017, the overall trophic index of the reservoir water was below 30, indicating a state of poor nutrition, clear water, and sufficient oxygen in the mean temperature layer.

## 4. Discussion

### 4.1. Synopsis of Temporal Variation of Chlorophyll-a Concentration

The concentration of chlorophyll-a in the Guanting Reservoir decreased significantly from 2016 to 2017. The study of the Guanting Reservoir by Peng et al. also showed that the Guanting Reservoir was in a comprehensive improvement stage in 2016–2017 [34]. The water quality pollution of Guanting Reservoir in 2019 has obvious regional characteristics. The water quality is good in the outbound area but is poor in the inbound area. It is consistent with the research of Yang et al. [57].

The study found that the chlorophyll-a concentration of the Guanting Reservoir increased in 2019. The increase of chlorophyll-a concentration in 2019 is related to season and indicates a potential eutrophication risk. According to research by Du et al., the algae in Guanting Reservoir grew vigorously and showed an increasing trend from April to September [58]. September is the best season for algae growth and reproduction, so the concentration of chlorophyll-a in September 2019 was higher than that in July 2017. Meanwhile, Guanting Reservoir in September is the wet season, and May is the dry season. When Chen et al. studied the small reservoirs in Nanjing, the chlorophyll-a concentration in the wet season was higher than that in the dry season [59]. Therefore, the increase of chlorophyll-a concentration in Guanting Reservoir in 2019 is the same as this rule.

### 4.2. Driving Factors of Chlorophyll-a Concentration Change

#### 4.2.1. Concentration of Chlorophyll-a in Recharge Flow

Figure 5 shows that because of the high chlorophyll-a concentration of the Guishui River in the northern reservoir, the inflow of the Guishui River into the reservoir influences its water quality. The chlorophyll-a concentration of the Guishui River gradually decreased, and the chlorophyll-a content of the northern reservoir also gradually decreased. Concurrently, the chlorophyll-a content at the entrance of the Yongding River is small and decreases annually, which indicates that the governance of the Yongding River is influential and that the water quality is better. Its water supply can improve the water quality of the reservoir through dilution.

#### 4.2.2. Flow Changes

**Figure 7:**
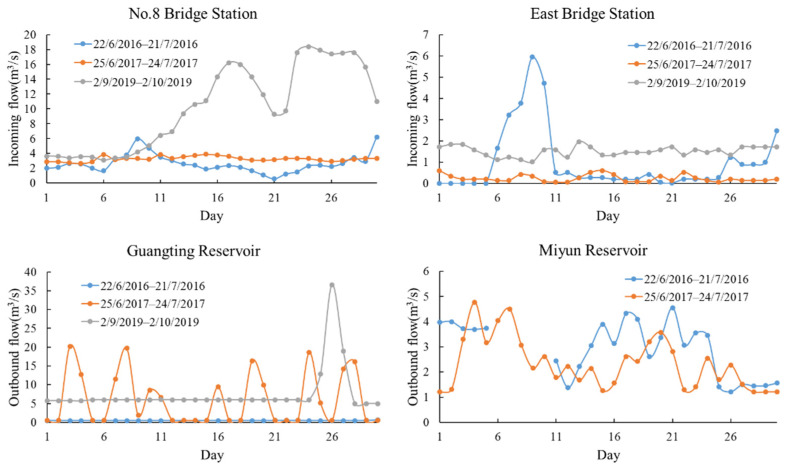
Inflow and outflow of the Guanting Reservoir (number 8 bridge measuring point and east bridge measuring point) 30 days before and after the retrieval date. Compared with Miyun Reservoir in the same period in 2016 and 2017, the annual change of the outbound flow of Guanting reservoir is larger.

We observed the statistical data of the daily inflow and outflow of the Guanting Reservoir within 30 days before and after the retrieval date of chlorophyll-a and found that the change was consistent with the change of chlorophyll-a concentration distribution in corresponding reservoirs (Figure 7). The number 8 bridge of the Guanting Reservoir is the monitoring point of the Yongding River. The inflow and outflow in 2016 were smaller than those in other years, and the chlorophyll-a concentration was generally higher. The inflow and outflow in 2019 gradually increased before and after the retrieval date, which lowered the chlorophyll-a concentration in the southern reservoir, especially at the entrance and exit close to 0 μg/L. The inflow in 2016 and 2017 was relatively stable, and the chlorophyll-a concentration decreased annually. However, the flow near Dongdaqiao station experienced a sudden increase and decrease for 5 days before 7 July 2016, which caused the chlorophyll-a concentration in the northern reservoir to gradually decrease because of the inflow of the Guishui River. The output flow of the Guanting Reservoir fluctuated greatly in the 30 days before and after the retrieval date in 2017. Therefore, in July 2017, the discharge of the Guanting Reservoir was extremely unstable, resulting in a low concentration of chlorophyll-a in the reservoir, with an average concentration of 0.77 μg/L.

#### 4.2.3. Water Quantity Changes

**Figure 8:**
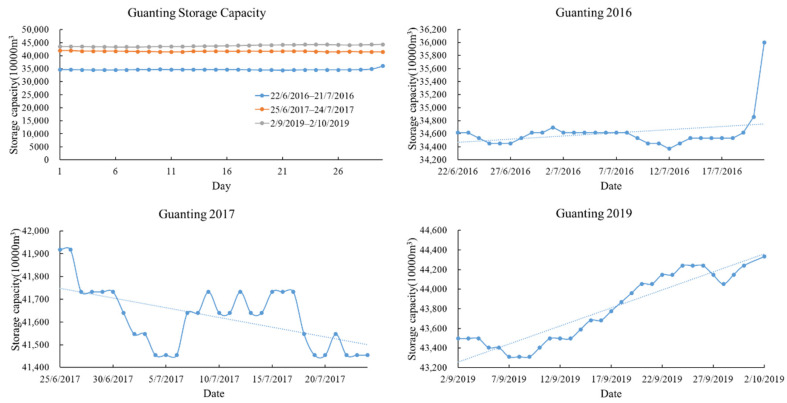
Water storage of the Guanting Reservoir within 30 days before and after retrieval date. From 2016 to 2017, the capacity of the reservoir increased. It had the largest capacity in 2019. From 25 June to 24 July, 2017, the daily variation of the reservoir capacity is large.

After 2016, the water storage of the Guanting Reservoir increased year by year (Figure 8), and the chlorophyll-a concentration decreased year by year. In general, the content of chlorophyll-a decreased with the increase of water surface area. In 2016, the change in a month before and after the retrieval date was small, and the water volume was stable and minimum, so the chlorophyll-a concentration was higher than that in 2017 and 2019. In 2017, the daily water storage of the Guanting Reservoir changed greatly, so the chlorophyll-a concentration was lower than that in 2016.

#### 4.2.4. Other Nutrients’ Changes

Chlorophyll is an important indicator of phytoplankton standing crop, while nitrogen and phosphorus are essential nutrients for phytoplankton growth [58]. Peng et al. Monitored the number 8 bridge measuring point of Guanting reservoir (located in Guishui River Reservoir Area) from 2016 to 2017. Except for total phosphorus, other monitoring indexes did not exceed the standard, which proved that the water quality was basically improved [34].

### 4.3. A Preliminary Attempt at Mutual Application of Chlorophyll-a Estimation Models in Adjacent Reservoirs

**Figure 9:**
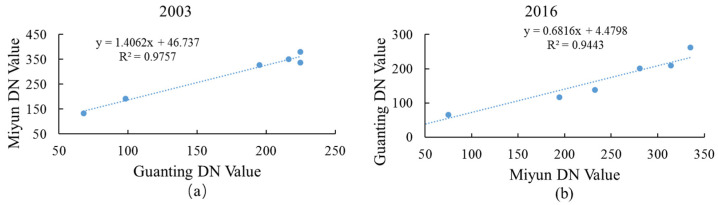
Estimates of regression curves of standard deviation of Landsat image bands’ DN values in Guanting Reservoir and Miyun Reservoir. The value of Guanting reservoir was used as a dependent variable to match the value of Miyun reservoir in 2003 (**a**). The value of Miyun reservoir was used as the dependent variable to match the value of Guanting reservoir in 2016 (**b**).

Miyun Reservoir (40°29′0″ N–40°30′5″ N, 116°50′0″ E–117°3′5″ E) is in the north of Beijing, China. By analyzing the reflectance of each band in the Landsat images of the Guanting and Miyun reservoirs, it was found that the standard deviation of each band had a high correlation (Figure 9). Through the linear regression model (Figure 9b), the band values of the Guanting Reservoir were calculated to the same magnitude as that of the Miyun Reservoir. Therefore, the estimation model of the Guanting Reservoir applied to the Miyun Reservoir can explore its changes. Based on the measured data of the Miyun Reservoir, Wang et al. used an empirical model to study the chlorophyll-a concentration of the Miyun Reservoir on 25 May 2003 [60]. Combined with Figure 9a, the distribution of chlorophyll-a concentration in 2003 can also be obtained.

**Figure 10:**
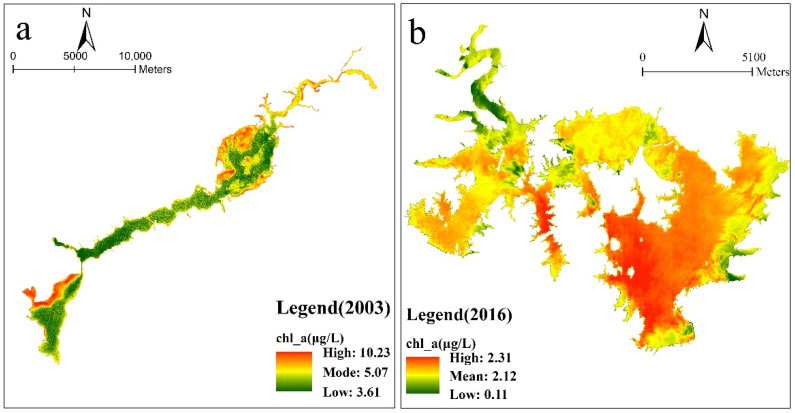
The distribution of chlorophyll-a concentration in Guanting obtained through the estimation model of Miyun Reservoir in 2003 (**a**). The distribution of chlorophyll-a concentration in Miyun obtained through the estimation model of Guanting Reservoir in 2016 (**b**). The color bar indicates the closeness of the concentration value to the mean. Red indicates values exceeding the mean, while green indicates values below the mean.

Yuan studied the nutritional status of Guanting Reservoir from January 2003 to June 2014 [61]. The monthly average values of two monitoring points (Gui1018 + 1: 40.38347° N, 115.779532° E; Gui Bridge: 40.335198° N, 115.69034° E) in the reservoir area recharged by Guishui River were 5.42 and 6.3 μg/L, respectively [56]. In 2003, the water surface area was small, and the total chlorophyll-a concentration was high. The concentration of chlorophyll-a was higher in the eastern reservoir but lower in the boundary and the Chaobai River inflow area (Figure 10a). According to the Miyun Reservoir estimation model, the values of Guanting Reservoir in 2003 were 5.17 and 4.82 μg/L, respectively. The retrieval results are relatively close to the onsite data of a previous study. According the research of Luan [62], in 2016, due to the severe excess of ammonia nitrogen and total nitrogen in the Chaohe River, the eastern area of Miyun Reservoir had a higher level of nutrition than the western area (Figure 10b), which was also consistent with the retrieval results. It was preliminarily proved that the method is feasible, and more efforts should be made to explore and validate in the future study.

### 4.4. Description of Chlorophyll-a Concentration Retrieval Method

The key to the empirical analysis method used in this article is to obtain accurate measured data through many field samples. The semi-analytical method is a method based on the optical characteristics of the water composition combined with a statistical model method or an empirical model method. The precise calculation process requires a large amount of data parameters. Machine learning is to train a model by using data, and it also requires a lot of data for training. Therefore, the empirical model is more effective when the measured data is single and limited. Reservoirs are closely related to human production and life. When there are accurate measurement data, the water quality distribution of the reservoir can be obtained quickly by this method, to explore the causes and manage it. The water quality retrieval based on the measured data proved successful, however, it does not give full play to the flexibility of remote sensing technology [63].

In the calibration and verification of the model, we found that the best band combination of each model included the blue band (450~515 nm) and the near-infrared band (845~885 nm). This may be because the reflectivity of these two bands is more sensitive to changes in the concentration of chlorophyll-a and can better distinguish the concentration of chlorophyll-a in the water. However, if only the blue and near-infrared bands are used for retrieval, the correlation is not ideal.

## 5. Conclusions

In this study, the concentration and distribution of chlorophyll-a in the Guanting Reservoir was estimated using a stepwise regression model. From 2016 to 2017, the chlorophyll-a concentrations of the reservoir decreased significantly, but in 2019, the chlorophyll-a concentration increased. We analyzed the reasons and found that the concentration of chlorophyll-a, the stability of water flow, the quantity of water, and the concentration of nitrogen and phosphorus are important factors affecting the concentration of chlorophyll-a in the reservoir. Through a comparative study of Landsat-8 OLI and Sentinel-2A data, we found that, according to the measured data, choosing the appropriate resolution can better retrieve the changes in water quality parameters.

The research results provide the basis and some valuable suggestions for reservoir management, especially for the relevant departments in Beijing, China. The concentration of chlorophyll-a in the Guanting Reservoir was well-controlled. However, due to the growth in 2019, the relevant departments cannot relax management. The improvement of the water quality of the Guanting Reservoir is still a focus. The control of the chlorophyll-a concentration in the inflow river is particularly important for reservoir water quality. In addition, the water quality of the reservoir can be improved by regulating the reservoir capacity.

Based on the correlation between the pixel values of two reservoirs in the same image, we tried to apply the estimation model from one reservoir to another reservoir. The method was verified by the average measured value of chlorophyll-a concentration in the Guanting Reservoir in 2003 and the distribution of nitrogen and phosphorus in Miyun Reservoir in 2016, which preliminarily proved that the method is feasible. We will continue to demonstrate this in the future.

The empirical retrieval method of water quality parameters is unstable and highly dependent on the measured data, which remains unsolved in this study. If conditions permit, the use of high-resolution remote sensing data may increase the detail of the retrieval. Follow-up research should further explore and verify these findings in more regions. Moreover, other commonly used retrieval methods should be explored.

## Figures and Tables

**Table 1:** Landsat and Sentinel image IDs. Timing is referenced to the Greenwich Mean Time (GMT).

ID	Path	Row	Date	Time
L71123032_03220030525	123	32	25/5/2003	/
LC08_L1TP_123032_20160707_20170323_01_T1	123	32	7/7/2016	2:53:24
LC08_L1TP_123032_20160621_20170323_01_T1	123	32	21/6/2016	2:53:16
LC08_L1TP_123032_20170710_20170725_01_T1	123	32	10/7/2017	2:53:19
LC08_L1TP_123032_20190918_20190926_01_T1	123	32	18/9/2019	2:53:48
S2A_MSIL1C_20160702T031632_N0204_R075_T50TLK_20160702T031629	204	75	2/7/2016	/
S2A_MSIL1C_20170707T031631_N0205_R075_T50TLK_20170707T031626	205	75	7/7/2017	/

**Table 2:** Description of the selected sample points. Effective sampling points refer to the number of abnormal points removed.

Year	Date	Effective Sampling Point	Data
2016	6 and 7 July	40	Chl-a
2017	10–12 July	38	Chl-a
2019	6 and 7 September	41	Chl-a

**Table 3:** Correlation coefficient (R) between measurements from 29 samples in the Guanting Reservoir and pixels’ value from Landsat-8 at corresponding time. The acquisition dates are 2016, 2017 and 2019, and the 22 variables include bands 1 to 7, ratio bands (b5/b4, b5/b3, b5/b2, b5/b1, b4/b3, b4/b2, b4/b1, b3/b2, b3/b1, b2/b1) and other indices (NDVI, SABI, KIVU, Apple, 3-band). Note that values with high correlation are shown in bold. The overall correlation of multiple band combinations is high.

**Year**	**b1**	**b2**	**b3**	**b4**	**b5**	**b6**	**b7**	**b5/b4**	**b5/b3**	**b5/b2**	**b5/b1**
2016	0.03	−0.03	−0.04	0.03	**0.73**	0.13	0.11	**0.74**	0.69	**0.72**	**0.73**
2017	−0.57	−0.67	−0.44	0.27	**0.77**	0.64	0.63	**0.79**	**0.80**	**0.82**	**0.82**
2019	−0.40	−0.44	−0.63	**−0.66**	−0.48	0.09	0.05	0.43	0.00	−0.21	−0.41
**Year**	**b4/b3**	**b4/b2**	**b3/b1**	**b3/b2**	**b3/b1**	**b2/b1**	**NDVI**	**SABI**	**KIVU**	**Apple**	**3-Band**
2016	0.11	0.12	0.02	−0.03	−0.08	−0.16	0.62	**0.74**	−0.10	**0.84**	0.42
2017	**0.78**	**0.74**	0.60	0.04	−0.21	−0.58	**0.79**	**0.78**	−0.74	0.71	**0.84**
2019	−0.61	**−0.75**	**−0.73**	−0.52	−0.70	−0.41	0.43	**0.75**	**0.74**	0.51	−0.32

**Table 4:** Comparison of correlation, exponential, logarithmic, polynomial, and power function models regarding best parameters in each year, and stepwise regression model. The correlation between the simulated value from the stepwise regression model and the measured value from samples is the highest.

Method	2016	*R*	2017	*R*	2019	*R*
Single band	*y* = 348.16 × (b5) + 202.5	0.74	*y* = 1123.2 × (b5) − 260.07	0.77	*y* = −0.0035 × (b4) + 3.24	0.66
	*y* = 861.2ln(b5) + 350.53	0.71	*y* = 499.43ln(b5) + 654.63	0.74	*y* = −1.84ln(b4) + 12.93	0.62
	*y* = −123.74 × (b5)^4^ + 1163.3 × (b5)^3^ − 3591.1 × (b5)^2^ + 4857.1 × (b5) − 1780.1	0.74	*y* = 641,228 × (b5)^4^ − 1 × 10^6^ × (b5)^3^ + 874,476 × (b5)^2^ − 271,000 × (b5) + 31,067	0.87	*y* = 2 × 10^−9^ × (b4)^4^ − 6 × 10^−6^ × (b4)^3^ + 0.0048 × (b4)^2^ − 1.81 × (b4) + 256.31	0.79
	*y* = 497.99 × (b5)^0.83^	0.66	*y* = 804.02 × (b5)^1.63^	0.76	*y* = 871566 × (b4)^−2.13^	0.65
	*y* = 443.88e^0.32(b5)^	0.64	*y* = 40.59e^3.65(b5)^	0.79	*y* = 12.163e^−0.004×(b4)^	0.7
Ratio of two bands	*y* = 0.46 × (b5/b4) + 0.29	0.74	*y* = 2.92 × (b5/b1) − 0.71	0.82	*y* = −4.32 × (b4/b2) + 5.92	0.75
	*y* = 1.13ln(b5/b4) + 0.48	0.72	*y* = 1.32ln(b5/b1) + 1.68	0.79	*y* = −4.68ln(b4/b2) + 1.61	0.74
	*y* = −0.08 × (b5/b4)^4^ + 0.62 × (b5/b4)^3^ − 1.04 × (b5/b4)^2^ + 0.29 × (b5/b4) + 1.19	0.75	*y* = 1294.3 × (b5/b1)^4^ − 2526.9 × (b5/b1)^3^ + 1822.8 × (b5/b1)^2^ − 572.82 × (b5/b1) + 66.47	0.87	*y* = 4296.6 × (b4/b2)^4^ − 19,098 × (b4/b2)^3^ + 31745 × (b4/b2)^2^ − 23,391 × (b4/b2) + 6449.9	0.82
	*y* = 0.6721 × (b5/b4)^0.82^	0.65	*y* = 2.35 × (b5/b1)^1.86^	0.81	*y* = 1.76 × (b4/b2)^−5.57^	0.79
	*y* = 0.6e^0.32(b5/b4)^	0.64	*y* = 0.08e^4.1(b5/b4)^	0.84	*y* = 308.51e^−5.17(b4/b2)^	0.81
Multi-band	*y* = 526,130 × (Apple) − 943,213	0.84	*y* = 1.18 × (3-band) − 0.61	0.84	*y* = 14.29 × SABI + 5.94	0.75
	*y* = −30,870 × 1(Apple)^4^ + 3 × 10^6^ × (Apple)^3^ − 9 × 10^6^ × (Apple)^2^ + 1 × 10^7^ × (Apple) − 6 × 10^6^	0.87	*y* = 390.55 × (3-band)^4^ − 762.22 × (3-band)^3^ + 551.62 × (3-band)^2^ − 174.28 − (3-band) + 20.067	0.91	*y* = 406,947 × (SABI)^4^ + 547,891 × (SABI)^3^ + 275,784 × (SABI)^2^ + 61,527 × (SABI) + 5135.8	0.85
	*y* = 1E + 06ln (Apple) − 670,142	0.87	*y* = 0.5288ln(3-band) + 0.352	0.81	*y* = 256.7e^16.46(SABI)^	0.77
Stepwise regression	*y* = 2.3 + 0.0000016 × Apple − 0.003 × b7	0.89	*y* = 1.46 + 1.17 × (3-band) − 0.002 × (b1) − 0.26 × (b5/b4)	0.95	*y* = 53.01 − 51.93 × (b4/b2) − 75.23 × KIVU − 2.05 × ((1/b4 − 1/b5) × b6)	0.92

**Table 5:** Verification of stepwise regression method for determination of chlorophyll-a concentration based on Landsat-8 OLI in the Guanting Reservoir. The *R*, *RMSE*, and *RRMSE* between the retrieval value from the stepwise regression model and the measurements from samples.

Date	*R*	*RMSE*	*RRMSE*	Number of Samples
July 2016	0.66	0.72	33%	11
July 2017	0.72	0.19	40%	9
September 2019	0.7	0.25	15%	12

**Table 6:** The stepwise regression model with the highest correlation between simulated values and measured values based on Landsat-8 in 2016 and 2017, and Sentinel-2A in 2016, 2017, and 2019 in the Guanting Reservoir.

Image Type	Year	Stepwise Regression Model	*R*	Number of Samples
Landsat-8	2016	*Y* = 2.304 + 0.0000016 × Apple − 0.003 × b7	0.89	29
	2017	*Y* = 1.457 + 1.167 × (3-band) − 0.002 × (b1) − 0.263 × (b5/b4)	0.95	29
	2019	*Y* = 53.01 − 51.928 × (b4/b2) − 75.229 × KIVU − 2.051 × ((1/b4 − 1/b5) × b6)	0.92	29
Sentinel-2A	2016	*Y* = 3.01 + 0.01 × B4 − 1.88 × (B4/b2) − 0.01 × B3	0.85	29
	2017	*Y* = 0.703 − 0.837 × (NDVI) + 0.0001 × b4	0.84	29

## Data Availability

The data presented in this study are available on request from the corresponding author.
